# Influence of anionic species on the low temperature pyrolysis performance of heated tobacco sheets catalyzed by sodium salts

**DOI:** 10.3389/fchem.2024.1425244

**Published:** 2024-06-12

**Authors:** Xuebin Zhao, Qiuling Wang, Dan Ai, Haiying Tian, Zhan Zhang, Ke Cao, Yixuan Wang, Wei Qi, Bo Li, Yapeng Niu, Lingchuang Meng, Beibei Gao, Bin Li

**Affiliations:** ^1^ China Tobacco Henan Industrial Co., Ltd., Zhengzhou, China; ^2^ Green Catalysis Center, College of Chemistry, Zhengzhou University, Zhengzhou, China

**Keywords:** low temperature, pyrolysis, heated tobacco sheets, sodium salts, anionic species

## Abstract

Development of low temperature catalytic pyrolysis technology for heated tobacco sheets is expected to increase the aroma of heated tobacco products and improve their overall smoking quality. In this study, the low temperature pyrolysis performances of heated tobacco sheets catalyzed by various anionic sodium salts were investigated using TG-DTG, Py-GC-MS technology and smoke routine chemical composition analysis. The results showed that the total weight loss between 100°C and 300°C increased by 7.8%–13.15% after adding various anionic sodium salts, among which, sodium acetate and sodium tartrate showed a relatively higher weight loss. The relative content of free hydroxyacetone, furfuryl alcohol, butyrolactone and megastigmatrienone in the pyrolysis gas increased, while the relative content of free nicotine decreased. With the change of anionic species, the catalytic decomposition ability of cellulose, lignin, and other substances may change, resulting in the distribution alteration of compounds in the pyrolysis gas. After adding sodium acetate and sodium citrate, the release of total particulate matter (TPM), glycerol, and nicotine in flue gas increased. Overall, the addition of sodium acetate and sodium citrate showed a higher low temperature pyrolysis performance of heated tobacco sheets. The research results in this paper provide data support for changing the low temperature catalytic pyrolysis performance of heated tobacco sheets by adjusting the type of anions in sodium salts.

## 1 Introduction

Heated tobacco products, which used closed heating instead of combustion to distill and release tobacco components, became an important new type of tobacco product due to their significantly reduced harmful substances and lack of smoldering (Enomoto et al., 2022; Watts et al., 2022; Wezyk-Caba et al., 2022; Yamamoto et al., 2022; Yuki et al., 2022; Kusonic et al., 2023; Vukas et al., 2023; Zhao et al., 2023). The heating temperature was generally around 300°C. However, due to the lower use temperature, heated tobacco sheets often failed to undergo pyrolysis reactions to produce flavor substances, resulting in lower smoke volume and aroma concentration, which significantly differed from traditional tobacco products (Eaton et al., 2018; Forster et al., 2018; Gasparyan et al., 2018; Li et al., 2023). Therefore, it is a crucial technical problem to develop low temperature pyrolysis technology for heated tobacco sheets, increasing the release of flavor in the development of heated tobacco products.

Catalysts are substances that can change reaction rates without changing the overall standard Gibbs free energy of the reaction (Uhe et al., 2011; Vojvodic and Norskov, 2015; Avanesian and Christopher, 2016; Bligaard et al., 2016; Campbell, 2017). Catalysts play a crucial role in minimizing the energy required for a reaction, thereby enabling it to take place under less extreme conditions and reducing energy consumption. The application of catalysts holds the potential to lower the energy barrier associated with tobacco sheet pyrolysis reactions, resulting in decreased pyrolysis temperature. This breakthrough would allow pyrolysis reactions, which traditionally necessitate high temperatures, to take place at lower temperatures. Consequently, a substantial quantity of flavor substances could be released during the process. Therefore, the development of low temperature catalytic pyrolysis technology for heated tobacco sheets is expected to be applied to increase the aroma of heated tobacco products and improve their overall smoking quality.

Currently, a variety of catalysts, including molecular sieves (Carlson et al., 2011; Martinez and Corma, 2011; Zhang et al., 2011; Dickerson and Soria, 2013; Li et al., 2014; Jia et al., 2017; Dai et al., 2020; Liang et al., 2021; Mardiana et al., 2022), mesoporous materials (Iliopoulou et al., 2007; Perego and Bosetti, 2011; Xu et al., 2012; Antunes et al., 2016; Herbst and Janiak, 2017; Shen et al., 2020), metal oxides (Barta et al., 2014; Matson et al., 2011; O'Neill et al., 2015; Robinson et al., 2016; Thompson et al., 2015), and alkali/alkaline earth metals (Xiong et al., 2021; Esso et al., 2022; Ge et al., 2022; Macedo et al., 2022; Wang et al., 2022; Ding et al., 2023), are widely employed in high temperature catalytic conversion processes for biomass. Heated tobacco sheets, as a unique type of biomass resource, have the potential to benefit from the application of these catalysts in low temperature catalytic pyrolysis. In our previous study (Li et al., 2023), we investigated the effects of different types of cations, such as sodium, potassium, magnesium, and calcium, on the low temperature pyrolysis performance of heated tobacco sheets. The results demonstrated that sodium catalysts significantly reduced the pyrolysis temperature and enhanced the release of flavor substances. Ding et al. (2017) conducted a study on the influence of organic potassium salts and inorganic potassium salts on the thermal decomposition performance of reconstructed sheets. They found that organic potassium salts had a more pronounced impact on the oxidation stage of coke at around 570°C compared to inorganic potassium salts. This suggested that the type of anion may also play a role in influencing the low temperature pyrolysis performance of tobacco sheets catalyzed by alkali metals.

In this article, various anionic sodium salts listed in the Chinese tobacco additive list were selected as catalysts. The low temperature pyrolysis performances of heated tobacco sheets were investigated using TG-DTG, Py-GC-MS, and regular chemical analysis of smoke. By examining the catalytic performance of different anionic sodium salts, this study provided data support for the potential application of low temperature pyrolysis technology to increase the aroma of heated tobacco sheets.

## 2 Experimental

### 2.1 Materials

The heated tobacco sheets used in this study were obtained from China Tobacco Henan Industry Co., Ltd., and their chemical composition can be found in the literature (Li et al., 2023). The reagents utilized in the experiment included the following high-quality substances: sodium acetate trihydrate (analytical grade, Tianjin Fuchen Chemical Reagent Factory), anhydrous sodium carbonate (analytical grade, Aladdin), anhydrous trisodium phosphate (analytical grade, Aladdin), sodium citrate (analytical grade, Aladdin), and sodium tartrate (analytical grade, Kemiou Chemical Reagent Co., Ltd.).

### 2.2 Loading experiments of various anionic sodium salts

Prior to conducting the loading test, the heated tobacco sheets were pulverized into fine powder. To impregnate the powder with different anionic sodium salts, a complete impregnation method was employed, with a fixed loading amount of 3% sodium. For instance, in the case of sodium acetate, 0.533 g of sodium acetate trihydrate and 3.0 g of water were accurately weighed and dissolved in a beaker. The resulting solution was then added to 3.0 g of tobacco sheet powder, thoroughly mixed, and sealed for 12 h to achieve equilibrium. Subsequently, the sample was subjected to drying at 100°C for 2 h to obtain the final product. The heated tobacco sheets samples after adding sodium acetate, sodium carbonate, trisodium phosphate, sodium citrate, and sodium tartrate were denoted as SA, SCA, SP, SC, and ST, respectively, by abbreviating the first letter of various anionic sodium salts. As a blank control, a sample containing only 3.0 g of water was prepared using the same procedure and designated as O.

### 2.3 Characterization of heated tobacco sheets loading with various anionic sodium salts

The sodium content in the tobacco sheet was detected using ICP-OES (Varian 720-ES), and the microstructure of the tobacco sheet sample was observed using SEM (ZEISS Sigma 500). The specific surface area and pore size distribution of the sample were determined using nitrogen physical adsorption (Micromeritics ASAP 2020), during which the specific surface areas of the samples were calculated using the BET formula, the total pore volumes were calculated based on the adsorption capacity of N_2_ when P/P_0_ is 0.98, and the pore diameter was calculated using the BJH method. The experimental parameters can be found in the literature (Li et al., 2023). The thermal properties of the samples were evaluated using a Mettler thermal analyzer. Typically, 3.00 mg of sample was placed in a crucible and heated at a rate of 10°C*min^−1^–500°C in a nitrogen atmosphere with a flow rate of 100 mL*min^−1^.

Py-GC-MS was performed by filling 5.00 mg of sample into the middle of a sample tube. The sample was first dried at 100°C for 60 s and then heated to 300°C at a rate of 20°C*s^−1^ and held for 20 s under a He atmosphere. The pyrolysis gas was analyzed by GC-MS (Agilent 7890A/5977A) during the heating process. The GC-MS parameters were as follows: the column temperature program started at 40°C for 3 min, then increased to 240°C at a rate of 5°C*min^−1^ and held for 3 min, and finally increased to 280°C at a rate of 20°C*min^−1^ and held for 32 min. The online injection mode with a split ratio of 10:1 was used, and the chromatographic column (DB-5ms) flow rate was set at 1 mL*min^−1^.

### 2.4 Analysis of conventional chemical components in the smoke of heated cigarettes

The HCI puffing mode was utilized to analyze of conventional chemical components in the smoke of heated cigarettes. The puffing parameters included a puff volume of 55 mL, a puff duration of 2 s, a puff interval of 30 s, and complete blockage of the ventilation holes. The release of smoke constituents, namely, total particulate matter (TPM), glycerol, and nicotine, was measured following the CORESTA Recommended Method No. 84. To calculate the per-puff release of smoke, software simulation was employed. This simulation allowed for the analysis of the measured data and the estimation of smoke constituent release on a per-puff basis. Factors such as puff volume, duration, interval, as well as the measured concentrations of TPM, glycerol, and nicotine in the smoke, were taken into account in the calculations.

## 3 Results and discussion

### 3.1 The effects of various anionic sodium salts on the physical and chemical properties of the heated tobacco sheets

**FIGURE 1:**
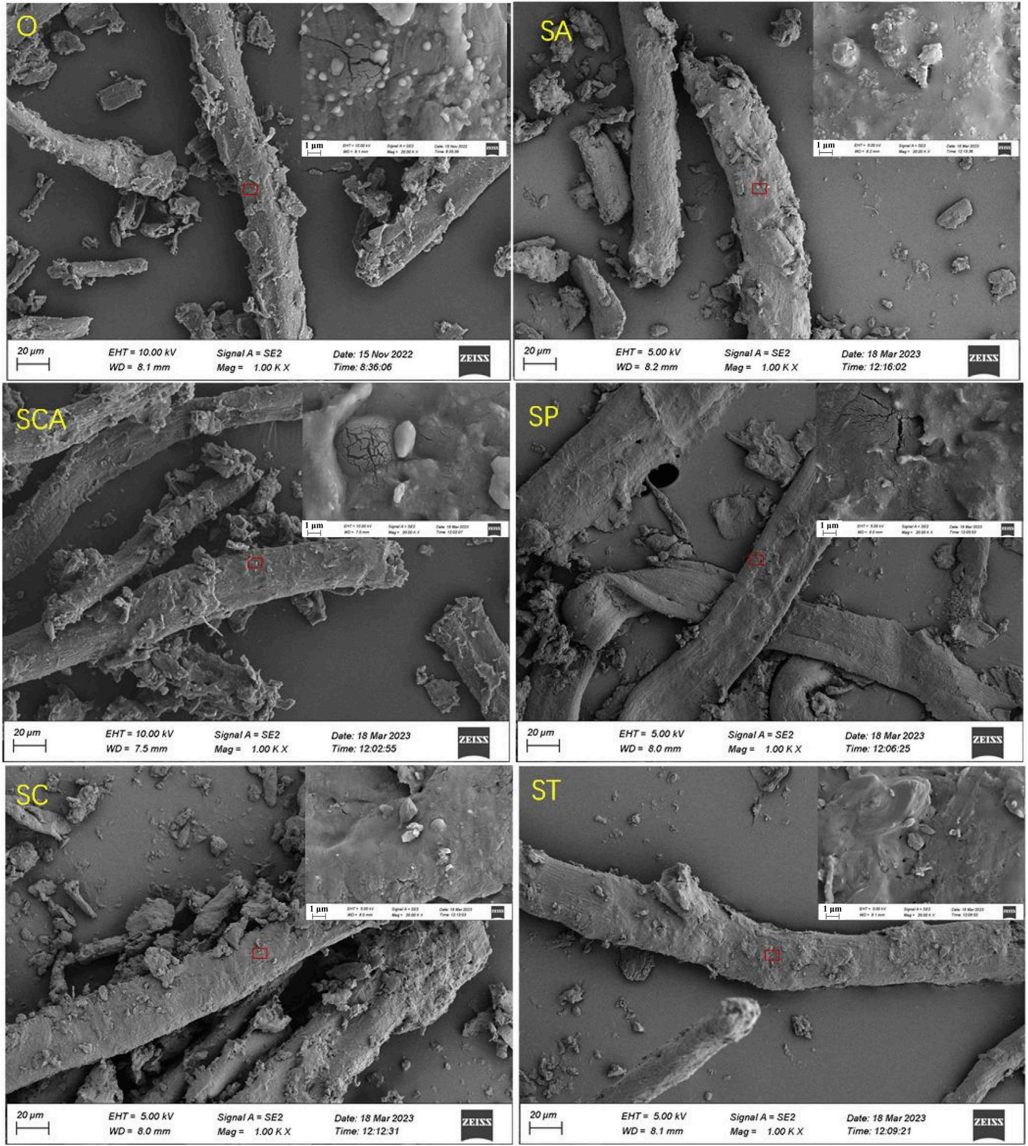
SEM images of heated tobacco sheets power before and after the addition of various anionic sodium salts.

The complete impregnation method was employed to introduce various anionic sodium salts to the sheet powder in this study, and the sodium content in the sheet samples were analyzed using ICP-OES. As presented in Table 1, the sodium content in the sheets ranged from 3.07% to 3.41% after the addition of various anionic sodium salts. The minimal difference in sodium content among the samples duly eliminated the influence of varying sodium contents, which also aligned with the experimental design of 3% sodium addition. Table 1 also displayed the changes in textural properties, such as specific surface area and pore volume of the samples. Following the addition of various anionic sodium salts, the specific surface area of the samples exhibited varying degrees of decrease. This suggested that some of the added sodium salts deposited on the surface of the samples, resulting in a reduction in specific surface area. Notably, the specific surface area experienced the greatest decrease after the addition of sodium carbonate. On the other hand, the pore volume and pore size increased to varying degrees after the addition of various sodium salts, indicating a significant pore expansion effect. The pore volume increased the most after adding sodium tartrate, while the pore size increased the most after adding sodium carbonate and sodium phosphate. Figure 1 illustrated the surface structure of the samples, which exhibited a fibrous structure. Further magnified images revealed that the sample surface was smooth, with micropores distributed. These observations suggested that the addition of various anionic sodium salts did not induce significant alterations to the surface structure of the samples. Consequently, the porous fiber structure was successfully retained.

**TABLE 1:** The sodium contents and texture properties of heated tobacco sheets before and after the addition of various anionic sodium salts.

Samples	Sodium contents (%)	Specific surface area (m^2^/g)	Pore volume (cm^3^/g)	Pore diameter (nm)
O	0.05	4.0205	0.0017	3.1132
SA	3.07	1.2301	0.0023	10.3440
SCA	3.22	0.6182	0.0025	27.0609
SP	3.11	2.0796	0.0085	21.8079
SC	3.41	3.6734	0.0018	13.1535
ST	3.38	1.8488	0.0231	4.4296

### 3.2 Thermogravimetric properties of heated tobacco sheets loaded with various anionic sodium salts

**FIGURE 2:**
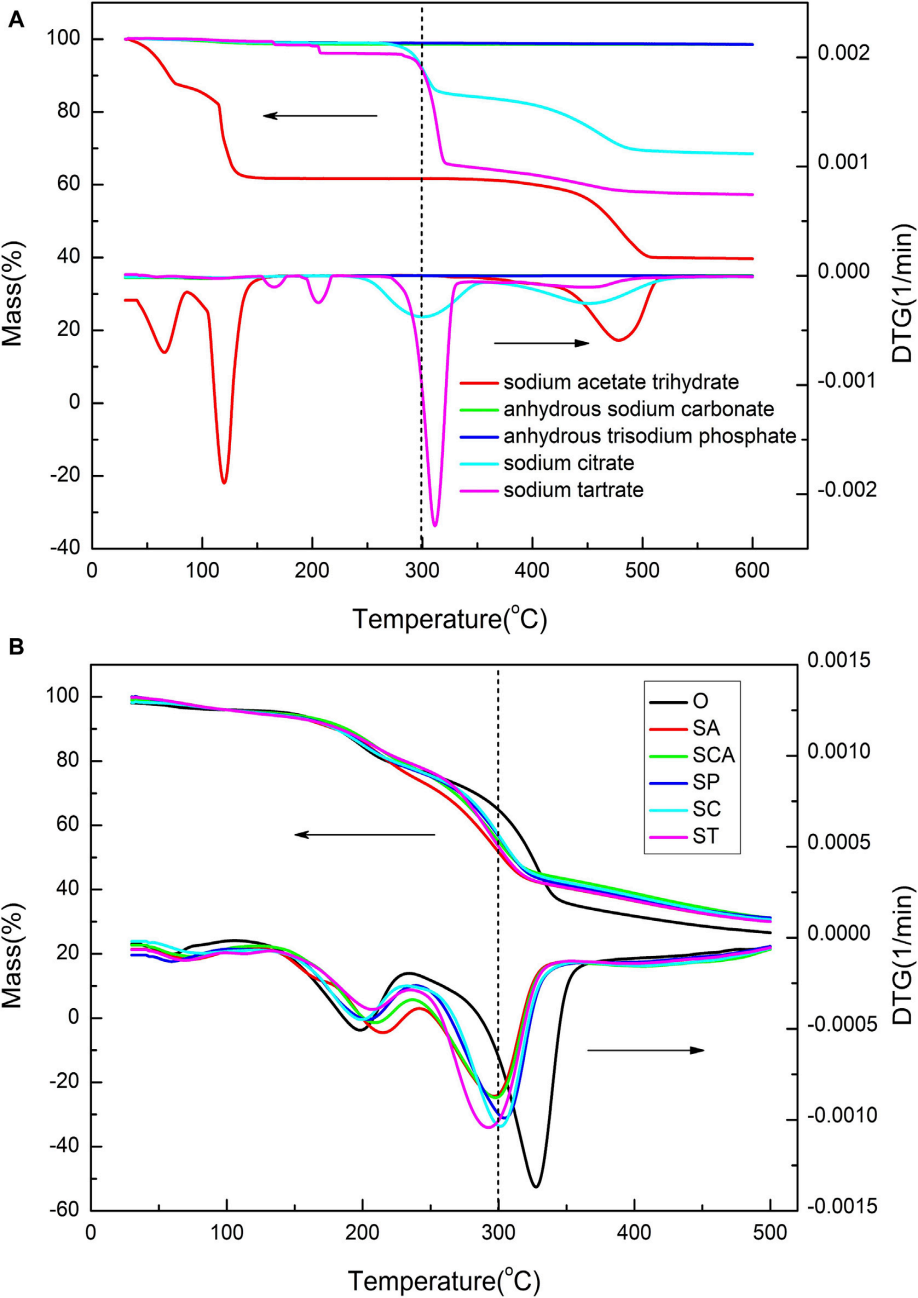
TG-DTG curves of various anionic sodium salts **(A)** and heated tobacco sheets before and after the addition of various anionic sodium salts **(B)**.

The thermal properties of the heated sheets before and after loading with various anionic sodium salts were analyzed using TG-DTG technology, and the results are shown in Figure 2B. In order to subtract the blank, the thermogravimetric curves of various anionic sodium salts are also presented in Figure 2A. For anhydrous sodium carbonate and anhydrous trisodium phosphate, there was almost no weight loss before 600 °C. However, sodium acetate trihydrate lost three crystalline water molecules before 150°C, and then remained relatively stable between 150 and 450°C. As for sodium citrate and sodium tartrate, there existed approximately 10% weight loss before 300 °C. Considering that the amount of sodium added to the heated tobacco sheets was 3%, the weight loss of around 10% before 300 °C had a minimal impact on the overall weight loss of the heated tobacco sheets. In summary, the weight loss of various anionic sodium salts themselves before 300 °C has a negligible effect on the weight loss of the heated tobacco sheets.

The TG curves of the samples loaded with various anionic sodium salts were significantly lower than that of the blank sample O at around 300 °C. This suggested that the weight losses of the samples loaded with various anionic sodium salts were higher than that of the blank sample at low temperatures. However, beyond 350°C, the TG curves of the samples loaded with various anionic sodium salts were considerably higher than that of the blank sample, indicating that the amounts of residual coke increased after the addition of sodium salts at high temperature. Therefore, the addition of sodium catalyst could significantly increase the weight loss of the sample at low temperature, but meanwhile it also leaded to a substantial increase in residual cokes at high temperatures.

The DTG curves of all samples revealed three distinct peaks, indicating that the weight loss process could be divided into three stages. The specific parameters for each stage of the samples were summarized in Table 2. In the case of sample O, the first stage occurred within the temperature range of 35–110°C, with a peak weight loss rate observed at 60 °C. This initial stage was primarily attributed to the evaporation of moisture present in the tobacco sheets, resulting in a weight loss of 2.08%. The second stage occurred between 110 and 235°C, with a maximum weight loss rate observed at 199 °C. The weight loss during this temperature range predominantly originated from the volatilization of humectants such as glycerol and propylene glycol, as well as a minor amount of nicotine evaporation. The cumulative weight loss during this stage amounted to 18.07%. The third stage occurred in the temperature range of 235–375°C, with the highest weight loss rate observed at 328 °C. This stage was primarily characterized by the release of volatile substances, including nicotine and flavoring agents, as well as the thermal decomposition of cellulose. The cumulative weight loss during this stage accounted for 44.49% of the total weight loss. Consequently, for sample O, the majority of weight loss was concentrated in the third stage, with the highest rate of weight loss observed around 328 °C.

**TABLE 2:** Three stages in TG-DTG curves of heated tobacco sheets before and after the addition of various anionic sodium salts.

Sample	The first stage				The second stage				The third stage				Loss between 100°C and 300 °C (%)
	T_s_/^o^C	T_f_/^o^C	T_m_/^o^C	Loss/%	T_s_/^o^C	T_f_/^o^C	T_m_/^o^C	Loss/%	T_s_/^o^C	T_f_/^o^C	T_m_/^o^C	Loss/%	
O	35	105	60	2.08	105	235	199	18.07	235	375	328	44.29	31.11
SA	41	115	74	4.02	115	242	215	21.45	242	348	296	33.28	44.26
SCA	41	110	75	3.27	110	237	209	17.66	237	347	298	34.48	40.95
SP	41	101	59	3.60	101	240	203	19.07	240	356	304	36.00	39.30
SC	41	122	82	3.23	122	233	199	16.70	233	356	301	36.57	38.91
ST	41	132	69	5.15	132	235	207	15.01	235	349	290	38.67	42.55

Note:T_s_: Start temperature, T_f_: Final temperature, T_m_: Maximum weight loss rate temperature.

Compared to sample O, the addition of various anionic sodium salts resulted in a slight increase in the maximum weight loss rate temperature during the second stage. However, the changes in weight loss were inconsistent. Specifically, the weight loss slightly increased when sodium acetate and sodium phosphate were added, while it slightly decreased with the addition of adding sodium carbonate, sodium citrate, and sodium tartrate. Furthermore, the addition of sodium salts led to a decrease in the maximum weight loss rate temperature during the third stage, which was consistent with previous research findings (Li et al., 2023). However, the extent of this decrease varied depending on the specific anion present in the sodium salt. The temperature decrease ranged from 24–38°C, with the ranking of the decrease as follows: ST > SA > SCA > SC > SP. These results indicated that the anions had a significant impact on the low temperature pyrolysis performance of heated tobacco sheets catalyzed by sodium salts.

Considering that the heating temperature of heated cigarettes is generally around 300 °C and since the majority of substances are released above 100°C, the total weight loss changes between 100 and 300°C was more relevant to understanding the changes in heated tobacco sheet performance in real-world usage scenarios. Although the total weight loss in the third stage decreased after adding sodium salts with various anions, compared to the blank sample, the total weight loss between 100 and 300°C increased after adding sodium salts with various anions. The increase ranged from 7.8% to 13.15%, with sodium acetate (SA) and sodium tartrate (ST) showing the highest increase. The ranking of the increase in total weight loss was as follows: SA > ST > SCA > SP > SC. These findings indicated that the addition of sodium salts with different anions at the heating temperature of cigarettes (300 °C) significantly increased the amount of smoke released, with sodium acetate and sodium tartrate having a more pronounced effect.

### 3.3 Analysis of free volatile components in pyrolysis gas of heated tobacco sheets loaded with various anionic sodium salts

**FIGURE 3:**
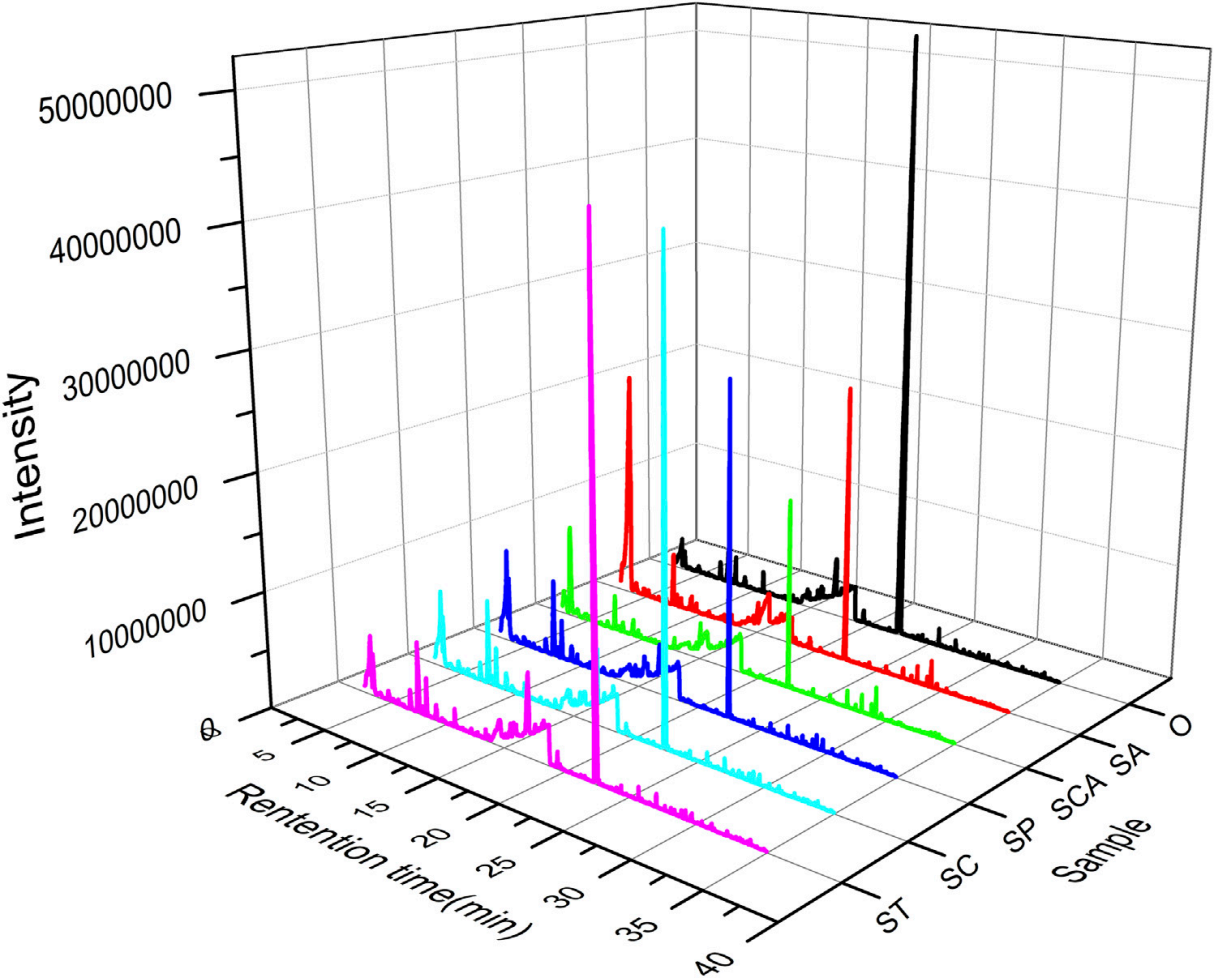
Py-GC-MS chromatogram of heated tobacco sheets before and after the addition of various anionic sodium salts.

Pyrolysis gas from heated tobacco sheets at 300 °C was analyzed using Py-GC-MS technology to investigate the volatile components. The obtained GC-MS spectrum was visually depicted in Figure 3, revealing notable variations in both the total peak area and total number of peaks with the introduction of various anions sodium salts. Upon comparison to the blank sample, it was observed that the total number of peaks increased while the total peak area decreased to varying extents following the addition of sodium salts with diverse anions. Particularly, the largest total peak area was observed when sodium tartrate, sodium citrate, and sodium acetate were incorporated into the heated tobacco sheets.

The volatile components in the pyrolysis gas were analyzed using GC-MS chromatography with a matching degree greater than 80 as the standard for qualitative analysis. Semi-quantitative analysis was performed based on peak area percentages (Li et al., 2023; Zhao et al., 2024), and the results are summarized in Table 3. In the blank sample, the main free volatile components in the pyrolysis gas were nicotine and glycerol. Additionally, small amounts of flavor substances such as hydroxyacetone, furfuryl alcohol, and coumarin were also detected. The introduction of various anions sodium salts had a significant impact on the relative content of free nicotine and glycerol. The relative content of free nicotine was observed to vary with the addition of different sodium salts with anions. The ranking of relative content of free nicotine was as follows: O > ST > SC > SP > SA > SCA. On the other hand, the relative content of free glycerol showed a different trend. The ranking of relative content of free glycerol was as follows: SCA > SP > ST > O > SC > SA. In summary, after the addition of sodium salts with various anions, the relative content of free nicotine showed a decrease to varying degrees. Conversely, the relative content of free glycerol increased after the addition of sodium carbonate, sodium phosphate, and sodium tartrate. However, it decreased after the addition of sodium citrate and sodium acetate.

**TABLE 3:** Quatitative results of pyrolysis gas of heated tobacco sheets loading different anionic sodium salts.

Rentention time (min)	Chemical compounds	Peak area percent (%)					
		O (%)	SA (%)	SCA (%)	SP (%)	SC (%)	ST (%)
3.57	Acetic acid	3.08	36.64	1.22	13.22	7.69	5.85
3.43	2-Propanone, 1-hydroxy-	1.08	1.41	10.44	3.90	2.60	2.08
4.07	Acetoin		0.59	0.98	0.16	0.12	0.10
4.60	1H-Pyrrole, 1-methyl-	0.11	0.10	0.22	0.06	0.03	0.01
5.494	Succindialdehyde			0.21			
6.183	3(2H)-Furanone, dihydro-2-methyl-					0.10	
6.83	2-Cyclopenten-1-one	0.924	0.45	0.40	0.85	0.89	0.94
7.55	2-Furanmethanol	1.77	2.50	2.22	4.81	4.47	3.45
8.24	4-Cyclopentene-1,3-dione	1.07	0.40	0.58	1.67	1.38	1.32
9.21	Butyrolactone		1.04	1.30	0.29	0.46	0.17
9.64	1,2-Cyclopentanedione		0.55	0.35	0.29	0.27	0.39
9.834	2-Cyclohexen-1-one		0.29	0.13	0.08	0.06	0.07
10.54	2-Furanmethanol, 5-methyl-		0.08	0.04	0.16	0.12	0.45
10.78	2-Furancarboxaldehyde, 5-methyl-	0.57	0.18	0.18	0.23	0.38	0.07
10.90	2-Butanone, 1-(acetyloxy)-	0.03	0.04		0.06	0.06	0.05
11.36	Isomaltol				0.03	0.04	
11.46	Phenol		0.04	0.23	0.06		
12.83	1,2-Cyclopentanedione, 3-methyl-		0.31	0.53	0.15		
12.998	2,5-Furandione, 3,4-dimethyl-					0.11	0.06
13.168	Benzyl alcohol			0.14	0.12		
13.453	Benzeneacetaldehyde			0.47	0.43	0.27	0.25
14.571	2-Pyrrolidinone			0.96			
14.75	Phenol, 2-methoxy-		0.16	1.10			
15.581	Maltol				0.30	0.34	0.35
16.09	1,2,3-Propanetriol, 1-acetate		13.03	9.02		4.55	
16.81	4H-Pyran-4-one, 2,3-dihydro-3,5-dihydroxy-6-methyl-(DDMP)		0.10		1.31	3.25	4.07
17.65	Glycerin	40.72	24.01	47.96	46.19	29.51	41.13
18.54	Catechol	0.02	0.17				
19.366	5-Hydroxymethylfurfural					0.32	0.53
20.18	1,2-Benzenediol, 3-methyl-		0.22	0.18	0.12	0.13	
20.89	Indole			0.05			
21.39	2-Methoxy-4-vinylphenol		0.08	0.09		0.11	
22.41	Pyridine, 3-(1-methyl-2-pyrrolidinyl)	48.88	11.10	7.19	15.29	27.67	30.88
23.628	Vanillin			0.02			
24.37	Pyridine, 3-(3,4-dihydro-2H-pyrrol-5-yl)-	0.12	0.11	0.20	0.20	0.17	0.16
27.05	2,3′-Dipyridyl	0.25	0.27	0.54	0.34	0.30	0.28
27.70	Megastigmatrienone	0.19	1.19	1.13	0.54	0.21	0.26
28.59	1,2,3,4-Tetrahydro-cyclopenta [b]indole		0.04				
32.20	Tetradecanoic acid	0.10			0.09	0.04	0.09
34.297	Pentadecanoic acid				0.04		0.04

In addition to free nicotine and glycerol, the addition of sodium acetate resulted in a significant increase in the content of free acetic acid in the pyrolysis gas. It was worth noting that although sodium acetate decomposed at approximately 400°C, even trace amounts of sodium acetate can contribute to the increased presence of free acetic acid in the pyrolysis gas. Therefore, the substantial rise in free acetic acid content can be attributed to the decomposition of trace amounts of sodium acetate. Furthermore, the addition of sodium phosphate, sodium tartrate, and sodium citrate also led to an increase in acetic acid content in the pyrolysis gas, although to a lesser extent. The presence of small amounts of acetic acid can be attributed to the pyrolysis of cellulose and other sugar substances, with sodium acting as a catalyst for their low temperature pyrolysis (Li et al., 2023).

Secondly, the relative content of intermediate products such as hydroxyacetone, furfuryl alcohol, and butyrolactone in the pyrolysis gas increased to varying degrees after the addition of different anions sodium salts. These intermediate products were formed during the pyrolysis of cellulose and other sugar substances (Antunes et al., 2016; Dai et al., 2020). Sodium had been identified as a catalyst for the low temperature pyrolysis of cellulose and other sugar substances (Li et al., 2023). Hence, the presence of different anions could regulate the distribution of low temperature pyrolysis products of cellulose and other sugar substances. This regulation could likely be attributed to the varying polarity of sodium in different anionic sodium salts, resulting in diverse catalytic abilities for the pyrolysis of cellulose. Finally, the addition of sodium salts with various anions resulted in significant changes in the content of phenol and methoxyphenol. Phenolic substances were commonly known as pyrolysis byproducts of lignin (Perego and Bosetti, 2011). This observation suggested that different anions could also impact the catalytic ability of sodium in lignin pyrolysis. Moreover, the relative content of megastigmatrienone in the pyrolysis gas of different samples exhibited significant variations.

**FIGURE 4:**
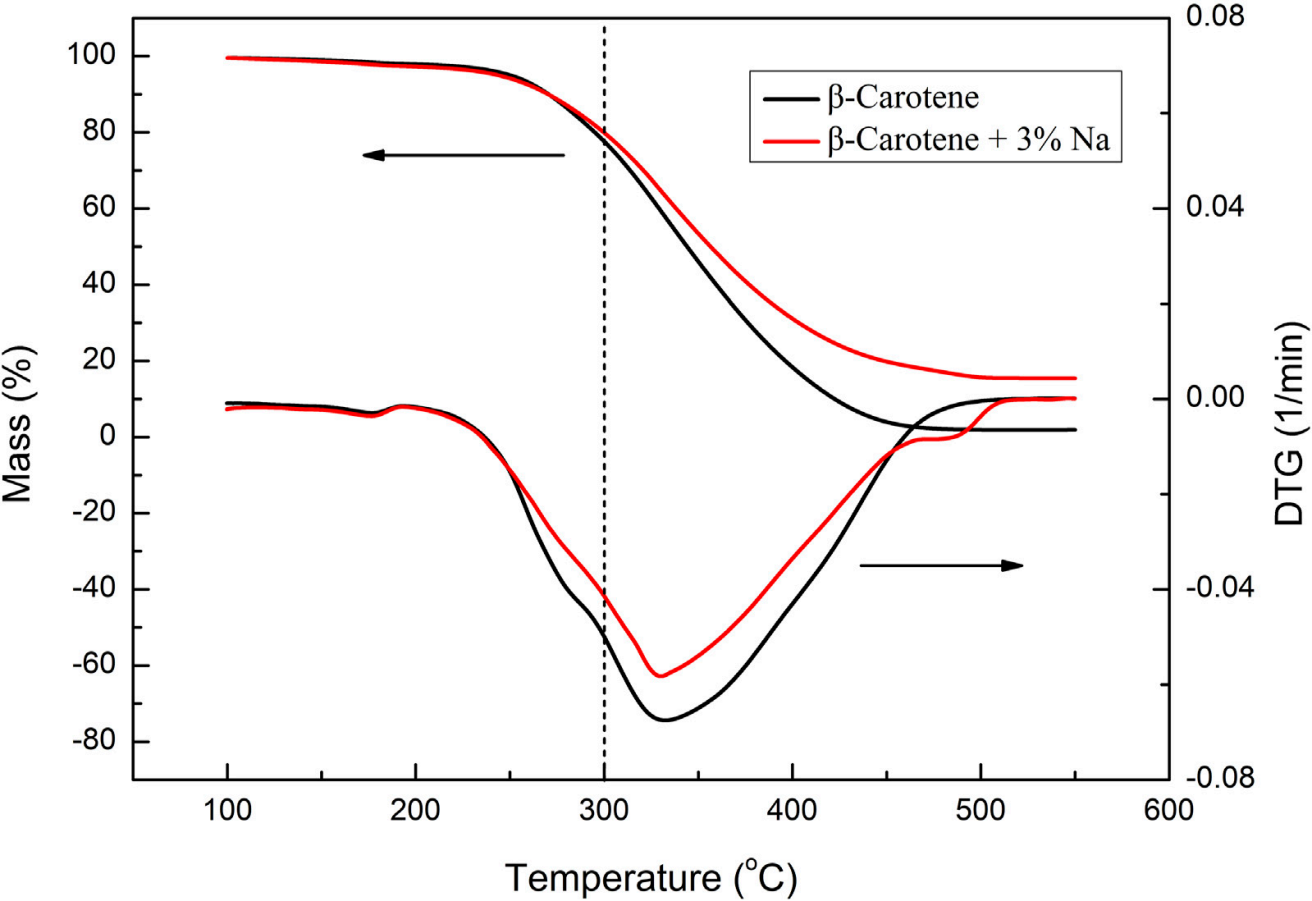
TG-DTG curves of β-carotene before and after the addition of 3% Na.

Moreover, as an important aroma substance, megastigmatrienone is naturally present in tobacco leaves and can also be generated through the pyrolysis of substances like carotenoids. The addition of various sodium salts led to an increase in the relative content of megastigmatrienone (Table 3). The alteration in megastigmatrienone content may be attributed to the catalytic pyrolysis of tobacco sheets by sodium salts, thereby disrupting the basic skeleton and altering the release amount of megastigmatrienone. Additionally, it was possible that different anionic sodium salts could modify the pyrolysis behavior of megastigmatrienone precursors and influence their release amount. To further confirm the reason for the increase in the content of megastigmatrienone, the low temperature pyrolysis performances of β-carotene catalyzed by of sodium salt were investigated as a model compound reaction. The thermogravimetric curve of β-carotene before and after the addition of 3% sodium were shown in Figure 4. After the addition of 3% sodium, the weight loss in the thermogravimetric curve of β-carotene between 100 and 300°C slightly decreased. This indicated that the addition of sodium salt catalyst cannot effectively catalyze the low temperature pyrolysis of megastigmatrienone precursors compounds. Therefore, the most likely reasons for the increase in megastigmatrienone in the pyrolysis gas of heated cigarette sheets after the addition of various anionic sodium salts were that sodium salt catalyzed the degradation of the tobacco sheets structure, which facilitated the release of megastigmatrienone presented in the tobacco sheets.

In conclusion, changing the type of anion in sodium salts could potentially modify its catalytic effect on the pyrolysis of substances such as cellulose and lignin, consequently leading to changes in the compound distribution within the pyrolysis gas. This discovery opened up the possibility of adjusting the chemical composition of the pyrolysis gas by manipulating the type of anion used.

### 3.4 The smoke conventional chemical composition analysis of heated cigarettes loaded with various anionic sodium salts

**FIGURE 5:**
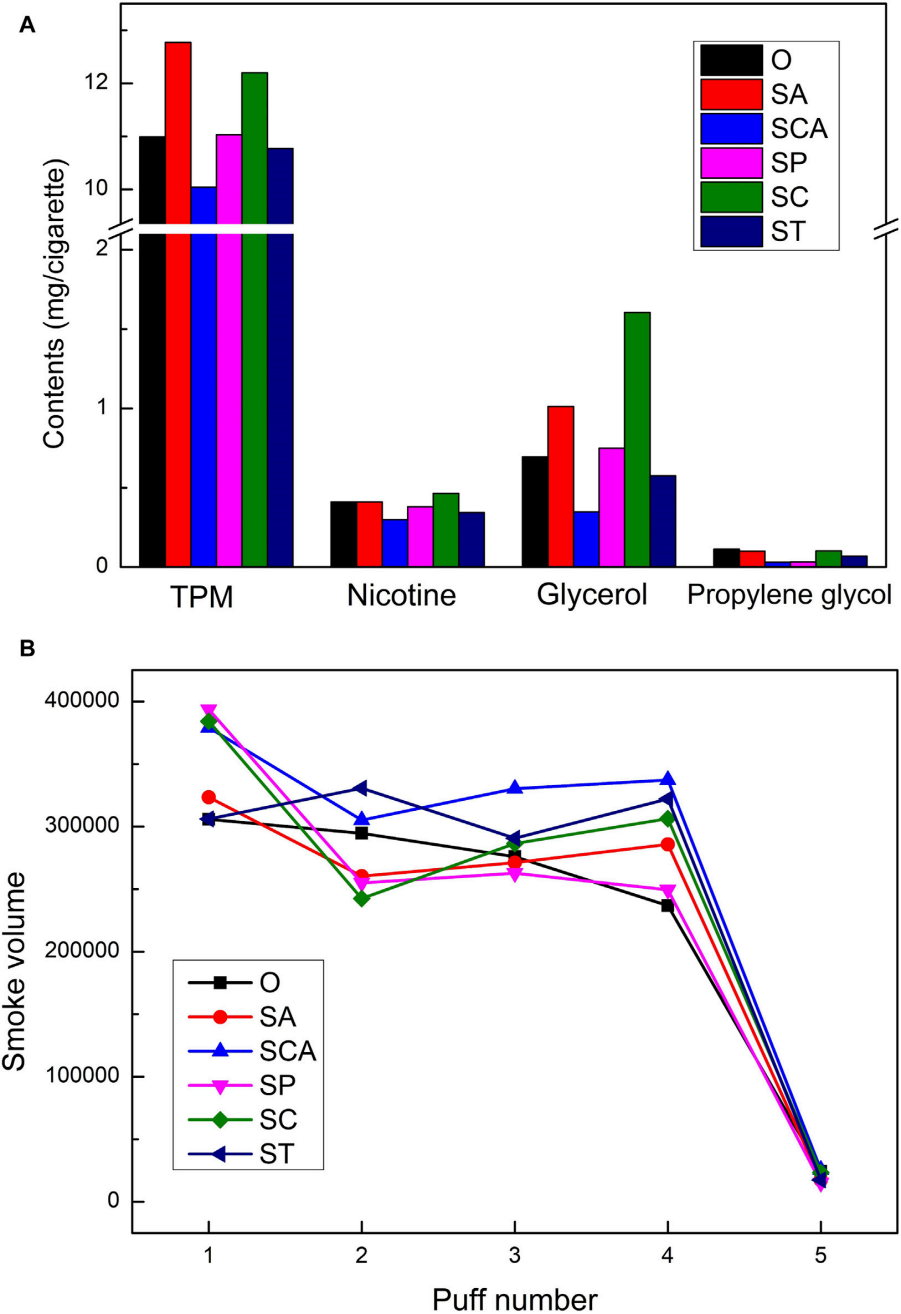
The conventional chemical composition **(A)** and the mouth-to-mouth release **(B)** of heated tobacco loaded with various anionic sodium salts.

Although Py-GC-MS technology was able to analyze the free volatile components in the pyrolysis gas, the smoke emitted from heated cigarettes was composed of both particulate matter and gas-phase matter, with the majority of the products being distributed in the particulate matter. Unfortunately, Py-GC-MS was not effective in analyzing the particulate matter. Therefore, in order to comprehensively investigate the effects of adding different anionic sodium salts on the smoke emitted from heated cigarettes, the changes in the conventional chemical composition of the smoke and the mouth-to-mouth release of the smoke were analyzed by using a smoking machine. The results are presented in Figure 5. Although the weight loss in the 100°C–300 °C range increased to varying degrees after the addition of virous anionic sodium salts, the changes in the conventional chemical composition of the smoke were inconsistent. The addition of sodium acetate and sodium citrate resulted in an increase in the release of TPM and nicotine in the smoke. On the other hand, the addition of sodium phosphate and sodium tartrate did not significantly affect the release of TPM, while the release of nicotine slightly decreased. The addition of sodium carbonate led to a slight decrease in the release of both TPM and nicotine. Furthermore, the addition of sodium acetate and sodium citrate significantly increased the release of glycerol, while the content of propylene glycol remained relatively unchanged. Conversely, the addition of sodium carbonate and sodium tartrate resulted in a decrease in the release of both glycerol and propylene glycol. Overall, the effect of increased content of conventional chemical composition after the addition of sodium acetate and sodium citrate was most pronounced, mainly showing an increase in the release of TPM, glycerol, and nicotine.

The simulation results of the mouth-to-mouth release of smoke showed in Figure 5B. For the blank sample, the mouth-to-mouth release of smoke gradually decreased with an increase in the number of puffs, and the smoke volume sharply decreased after the fifth puff. After adding various anionic sodium salts, the releasement of smoke in the first puff increased, and it increased significantly after adding sodium phosphate, sodium citrate, and sodium carbonate. Except for sodium tartrate, the mouth-to-mouth release of smoke after adding other anionic sodium salts showed a trend of decreasing first, then increasing, and then decreasing again, which was different from the decreasing trend of the blank sample. This may have been due to the gradual highlighting of the catalytic effect of different anionic sodium salts with an increase in the number of puffs, resulting in an increase in the release of smoke. Overall, the mouth-to-mouth release stability of smoke improved after adding various anionic sodium salts, especially when smoking the fourth puff, the release of smoke from all samples was higher than that of the blank sample. After adding sodium carbonate and sodium tartrate, the mouth-to-mouth release of smoke increased significantly.

## 4 Conclusion

The thermogravimetric results showed that the total weight loss of the thermogravimetric curve between 100 and 300°C increased after adding different anionic sodium salts, with an increase ranging from 7.8% to 13.15%. The weight loss between 100 and 300°C was significantly increased after adding sodium acetate and sodium tartrate. The results of pyrolysis gas analysis showed that the relative content of free hydroxyacetone, furfuryl alcohol, and butyrolactone, megastigmatrienone in the pyrolysis gas increased, while the relative content of free nicotine decreased. With the change of anionic species, the ability to catalyze the decomposition of cellulose, lignin, and other substances may change, resulting in a change in the distribution of compounds in the pyrolysis gas. The analysis of the conventional chemical components in the smoke showed that after adding sodium acetate and sodium citrate, the release of total particulate matter (TPM), glycerol, and nicotine in the smoke increased. The stability of the release of smoke with different anionic sodium salts added was also improved.

## Data Availability

The original contributions presented in the study are included in the article/supplementary material, further inquiries can be directed to the corresponding authors.
