# Corrigendum: Influence of anionic species on the low temperature pyrolysis performance of heated tobacco sheets catalyzed by sodium salts

**DOI:** 10.3389/fchem.2024.1508480

**Published:** 2024-11-07

**Authors:** Xuebin Zhao, Qiuling Wang, Dan Ai, Haiying Tian, Zhan Zhang, Ke Cao, Yixuan Wang, Wei Qi, Bo Li, Yapeng Niu, Lingchuang Meng, Beibei Gao, Bin Li

**Affiliations:** ^1^ China Tobacco Henan Industrial Co., Ltd., Zhengzhou, China; ^2^ Green Catalysis Center, College of Chemistry, Zhengzhou University, Zhengzhou, China

**Keywords:** low temperature, pyrolysis, heated tobacco sheets, sodium salts, anionic species

In the published article, there was an error in the **Conflict of Interest** statement. A past collaboration between the handling editor and four of the authors was not declared. The correct statement appears below:

“Authors XZ, QW, DA, HT, ZZ, KC, YW, WQ, BoL, YN, and BiL were employed by China Tobacco Henan Industrial Co., Ltd.

The remaining authors declare that the research was conducted in the absence of any commercial or financial relationships that could be construed as a potential conflict of interest.

The handling editor BL declared a past collaboration with the authors HT, ZZ, WQ and BoL.”

The authors would also like to add the following **Acknowledgments** statement:

“The authors would like to clarify that the co-author Bin Li is a researcher affiliated with China Tobacco Henan Industrial Co. Ltd., while the handling editor Bin Li is a different researcher affiliated with Zhengzhou Tobacco Research Institute of CNTC.”

The authors apologize for these errors and state that this does not change the scientific conclusions of the article in any way. The original article has been updated.

